# Comparing the association of GFR estimated by the CKD-EPI and MDRD study equations and mortality: the third national health and nutrition examination survey (NHANES III)

**DOI:** 10.1186/1471-2369-13-42

**Published:** 2012-06-15

**Authors:** Tariq Shafi, Kunihiro Matsushita, Elizabeth Selvin, Yingying Sang, Brad C Astor, Lesley A Inker, Josef Coresh

**Affiliations:** 1 Department of Medicine, Division of Nephrology, Johns Hopkins University School of Medicine, Baltimore, MD, USA; 2 Welch Center for Prevention, Epidemiology and Clinical Research, Johns Hopkins Medical Institutions, Baltimore, MD, USA; 3 Department of Epidemiology, Johns Hopkins Bloomberg School of Public Health, Baltimore, MD, USA; 4 Department of Medicine and Public Health, University of Wisconsin, Madison, WI, USA; 5 Department of Medicine, Division of Nephrology, Tufts Medical Center, Boston, MA, USA; 6 Department of Biostatistics, Johns Hopkins Bloomberg School of Public Health, Baltimore, MD, USA

**Keywords:** Glomerular filtration rate, Chronic kidney disease, Epidemiology, Outcomes

## Abstract

**Background:**

The Chronic Kidney Disease Epidemiology Collaboration equation for estimation of glomerular filtration rate (eGFR_CKD-EPI_) improves GFR estimation compared with the Modification of Diet in Renal Disease Study equation (eGFR_MDRD_) but its association with mortality in a nationally representative population sample in the US has not been studied.

**Methods:**

We examined the association between eGFR and mortality among 16,010 participants of the Third National Health and Nutrition Examination Survey (NHANES III). Primary predictors were eGFR_CKD-EPI_ and eGFR_MDRD_. Outcomes of interest were all-cause and cardiovascular disease (CVD) mortality. Improvement in risk categorization with eGFR_CKD-EPI_ was evaluated using adjusted relative hazard (HR) and Net Reclassification Improvement (NRI).

**Results:**

Overall, 26.9% of the population was reclassified to higher eGFR categories and 2.2% to lower eGFR categories by eGFR_CKD-EPI,_ reducing the proportion of prevalent CKD classified as stage 3–5 from 45.6% to 28.8%_._ There were 3,620 deaths (1,540 from CVD) during 215,082 person-years of follow-up (median, 14.3 years). Among those with eGFR_MDRD_ 30–59 ml/min/1.73 m^2^, 19.4% were reclassified to eGFR_CKD-EPI_ 60–89 ml/min/1.73 m^2^ and these individuals had a lower risk of all-cause mortality (adjusted HR, 0.53; 95% CI, 0.34-0.84) and CVD mortality (adjusted HR, 0.51; 95% CI, 0.27-0.96) compared with those not reclassified. Among those with eGFR_MDRD_ >60 ml/min/1.73 m^2^, 0.5% were reclassified to lower eGFR_CKD-EPI_ and these individuals had a higher risk of all-cause (adjusted HR, 1.31; 95% CI, 1.01-1.69) and CVD (adjusted HR, 1.42; 95% CI, 1.01-1.99) mortality compared with those not reclassified. Risk prediction improved with eGFR_CKD-EPI_; NRI was 0.21 for all-cause mortality (p < 0.001) and 0.22 for CVD mortality (p < 0.001).

**Conclusions:**

eGFR_CKD-EPI_ categories improve mortality risk stratification of individuals in the US population. If eGFR_CKD-EPI_ replaces eGFR_MDRD_ in the US, it will likely improve risk stratification.

## Background

Decreased kidney function is an independent risk factor for mortality. While measured glomerular filtration rate (GFR) remains the gold standard for assessing decreased kidney function, in routine clinical practice, GFR is usually estimated from serum creatinine by the Modification of Diet in Renal Disease (MDRD) Study equation (eGFR_MDRD_). Understanding the association of eGFR categories with clinical outcomes is an important aspect of the chronic kidney disease (CKD) staging system. Accurate classification of individuals with CKD can inform healthcare utilization and therapeutic decision making by reducing false positive diagnoses of CKD while correctly classifying those with CKD to appropriate risk categories [1].

The Chronic Kidney Disease Epidemiology Collaboration (CKD-EPI) equation for estimation of GFR from serum creatinine (eGFR_CKD-EPI_) improves GFR estimation compared with the MDRD Study equation [2]. eGFR_CKD-EPI_, as compared to eGFR_MDRD_, results in a lower prevalence of decreased eGFR [2]. Recent publications have demonstrated that use of the CKD-EPI equation results in reclassification of individuals previously classified using eGFR_MDRD_ to different eGFR_CKD-EPI_ categories with more appropriate risk stratification [3-6]. However, the effect of this reclassification from eGFR_MDRD_ categories by CKD-EPI equation on long-term risk prediction in a nationally representative sample of the US population has not been described. The objective of this study was to evaluate the effect of reclassification using eGFR_CKD-EPI_ on the estimated risk of all-cause and cardiovascular disease (CVD) mortality in the US population using data from the Third National Health and Nutrition Examination Survey (NHANES III) and relevant subgroups.

## Methods

### Study sample

NHANES III is a cross-sectional, multistage, stratified, clustered probability sample of the noninstitutionalized US civilian population conducted during 1988–1994 by the National Center for Health Statistics (NCHS), a branch of the Center for Disease Control and Prevention [7]. In NHANES III, certain subgroups of the population were oversampled including Mexican Americans, non-Hispanic blacks and elderly persons to ensure adequate sample sizes of these groups. We limited the study population to 16,010 persons aged 17 years or older who were examined at the mobile examination center (MEC), were not missing serum creatinine data or covariates of interest, and had available mortality follow-up. Mortality follow-up was available for 99.9% of the eligible participants. The protocols for conduct of NHANES were approved by the NCHS institutional review board and informed consent was obtained from all participants. Procedures were followed in accordance with ethical standards of the Johns Hopkins School of Public Health Office of Human Subjects Research and Institutional Review Board.

### Measurements

NHANES III procedures have been previously described [7]. Briefly, standardized questionnaires were administered at home and physical examination and laboratory tests specimen collection was performed at the MEC. Self-reported race/ethnicity was categorized as non-Hispanic White, non-Hispanic Black, Mexican-American or other. Smoking was defined as either active cigarette smoking, having smoked >100 cigarettes in life, or never having smoked. Participants were considered to have diabetes mellitus if they reported being told by a doctor that they had diabetes at a time other than pregnancy or if they were taking insulin or oral hypoglycemic agents. Cardiovascular disease (CVD) was considered to be present at baseline if the participant reported being informed by a doctor of prior heart attack, congestive heart failure or stroke. Antihypertensive medication use was based on self-report. Blood pressure (BP) was measured using standard techniques and reported as the average of all systolic and diastolic readings. Participants were advised to fast prior to specimen collection but fasting was not required. Overall, 87% of the participants had fasted for ≥ 6 hours prior to blood draw.

Serum creatinine was measured using a kinetic rate Jaffe method. Serum creatinine measurements were recalibrated to standardized creatinine measurements obtained at the Cleveland Clinic Research Laboratory (Cleveland, Ohio) as described previously [standard creatinine = (0.960 x serum creatinine) – 0.184] [8]. We calculated eGFR using the isotope dilution mass spectrometry (IDMS)-traceable 4-variable MDRD Study equation and the CKD-EPI equation [2,9]. There was no difference in eGFR_CKD-EPI_ based on fasting status (0.98 ml/min/1.73 m^2^ lower in those fasting ≥6 hours; p = 0.14). We categorized eGFR into the following clinically relevant categories: ≥120, 90–119, 60–89, 30–59 and <30 ml/min/1.73 m^2^. Within each category of eGFR_MDRD_, individuals were reclassified into three groups based on eGFR_CKD-EPI_: a) higher eGFR_CKD-EPI_ category; (b) same eGFR category by both eGFR_MDRD_ and eGFR_CKD-EPI_, and; (c) lower eGFR_CKD-EPI_ category.

C-reactive protein (CRP) was measured by latex-enhanced nephelometry (Dade Behring). CRP level was categorized as either undetectable by the assay (<0.22 mg/dL), minimal (0.22-0.99 mg/dL) or elevated (≥1.0 mg/dL). Urinary albumin level was measured by solid-phase fluorescence immunoassay, and urinary creatinine level was measured by the modified kinetic method of Jaffe using a Beckman Coulter Synchron AS/Astra Analyzer (Beckman Coulter, Inc., Fullerton, California). Albuminuria was expressed as urinary albumin-to-creatinine ratio (ACR) and categorized into 3 categories; <30 mg/g, 30–299 mg/g and ≥300 mg/g.

### Causes of death

Causes of death were obtained using the NHANES III Linked Mortality Public-use File [10]. This file contains mortality follow-up data on NHANES III participants obtained *via* National Death Index (NDI) linkage through December 31, 2006. Mortality ascertainment is performed using probabilistic matching between NHANES III and NDI using previously validated methods [10]. Cause of death coding for all U.S. deaths occurring prior to 1999 followed the 9th revision of the International Statistical Classification of Diseases, Injuries, and Causes of Death (ICD-9) guidelines, while all deaths after 1998 followed the 10th revision of the International Statistical Classification of Diseases, Injuries, and Causes of Death (ICD-10) guidelines. All deaths occurring prior to 1999 were recoded from ICD-9 to comparable ICD-10 (I) based cause of death groups [11]. We defined cardiovascular disease mortality using the following cause of death groups: 056 (hypertensive heart disease [I11]), 057 (hypertensive heart and renal disease [I13]), 058–063 (ischemic heart diseases [I20-I25]), 067 (heart failure [I50]), 068 (valvular heart diseases and cardiomyopathy [I26-I28, I34-I38, I42-I49, I51]), 069 (essential hypertension and hypertensive renal disease [I10, I12]), 070 (cerebrovascular disease [I60-I69]), and 071 (atherosclerosis [I70]).

### Statistical analysis

All analyses were performed using 6-year MEC sampling weights provided by the National Center for Health Statistics that account for the complex survey design of NHANES III as well as probabilities for non-response. Analyses were performed using survey (svy) commands Stata 10.1 and 11.2. (Stata Corp, http://www.stata.com) Baseline characteristics were compared across eGFR categories and reclassification status of the participants. Survival analysis techniques were used to analyze the risk of all-cause and CVD mortality. Individuals who were alive on December 31, 2006 were censored in the analyses. Modified Cox proportional hazards regression was used to model the risk of death across eGFR and reclassification categories. Hazard ratios (HR) were calculated to assess risk of death after adjustment for age, race/ethnicity, sex, body mass index, systolic and diastolic BP, antihypertensive medication use, history of diabetes and CVD, smoking status, serum total cholesterol, ACR categories and CRP categories. Poisson regression was used to calculate and display incidence rates with eGFR modeled as a restricted cubic spline with knots at 30, 45, 60, 90 and 120 ml/min/1.73 m^2^.

### Reclassification

To assess reclassification we calculated net reclassification improvement (NRI) [12]. Net reclassification improvement (NRI) is a statistic that allows calculation of the effect of reclassification of individuals from one disease category to the other. It is a difference of two ratios; clinically correct reclassification minus clinically incorrect classification. The range of this difference is from −1 to +1 with a negative number reflecting incorrect reclassification and a positive number indication correct reclassification.

For NRI calculations we excluded individuals with eGFR ≥120 ml/min/1.73 m^2^ by either equation as the high eGFR in this group reflects low serum creatinine likely from malnutrition, intercurrent illness and low muscle mass [3]. To calculate NRI, we first created cross-tabulation of participants in eGFR_MDRD_ and eGFR_CKD-EPI_ categories stratified by the outcome status (alive or dead). We then calculated the proportion of individuals in each category of eGFR_MDRD_ that are reclassified by eGFR_CKD-EPI_. Clinically correct reclassification was defined as: proportion of participants reclassified to higher eGFR category by eGFR_CKD-EPI_ among those who are alive + the proportion of participants reclassified to lower eGFR category by eGFR_CKD-EPI_ among those who died. Clinically incorrect reclassification was defined as: proportion of participants reclassified to higher eGFR category by eGFR_CKD-EPI_ among those who died + the proportion of participants reclassified to lower eGFR category by eGFR_CKD-EPI_ among those who are alive. NRI = clinically correct reclassification – clinically incorrect reclassification. Statistical significance for NRI was calculated using bootstrapping with replacement. To account for the confounding effect of age, sex and race on outcomes, we also calculated stratum-specific NRI in these subgroups.

## Results

### Baseline characteristics

The baseline characteristics of the participants stratified by reclassification status by eGFR_CKD-EPI_ are presented in Table1. Overall, the number of participants (population %) reclassified were as follows: 3,464 (26.9%) of the participants were reclassified to a higher eGFR category, 559 (2.2%) were reclassified to a lower eGFR category and 11,987 (70.8%) were not reclassified. There was only 1 participant with eGFR_MDRD_ <30 ml/min/1.73 m^2^ who was reclassified upward (data not presented in Table1). Individuals reclassified to higher eGFR_CKD-EPI_ categories were more likely to be younger, female, had lower prevalence of diabetes and CVD, and had lower BP, cholesterol, CRP and less albuminuria. These differences were much more pronounced at lower eGFR categories (<60 ml/min/1.73 m^2^). Among participants with eGFR_MDRD_ <120 ml/min/1.73 m^2^, no participants below 65 years were reclassified to a lower eGFR_CKD-EPI_ category.

**Table 1:** **Baseline characteristics of 16,010 NHANES III (1988–1994) participants stratified by eGFR**_**MDRD**_**and eGFR**_**CKD-EPI**_

**EGFR**_**MDRD**_	**≥120**		**90-119**			**60-89**			**30-59**			**<30**
**(No. of participants)**^**a**^	**(2,729)**		**(6,604)**			**(5,606)**			**(1,015)**			**(56)**
**EGFR**_**CKD-EPI**_	**≥120**	**90-119**	**≥120**	**90-119**	**60-89**	**90-119**	**60-89**	**30-59**	**60-89**	**30-59**	**<30**	**<30**
Number of participants ^a^ (%) ^b^	2,416	312 ^d^	1,473	4,969	162	1,870	3,664	72	120	883	12	55 ^e^
	(88.3)	(11.7)	(20.2)	(78.4)	(1.4)	(42.9)	(56.6)	(0.5)	(19.4)	(79.5)	(1.1)	(99.5)
**Characteristic**^c^												
Serum Creatinine (mg/dl)	0.64	0.59	0.76	0.79	0.70	0.87	0.94	1.07	1.07	1.26	1.97	2.76
	(0.004)	(0.01)	(0.003)	(0.002)	(0.01)	(0.004)	(0.004)	(0.03)	(0.02)	(0.02)	(0.11)	(0.20)
EGFR_MDRD_ (ml/min/1.73 m^2^)	138	131	112	100	98	85	74	61	58	49	31	23
	(0.63)	(0.72)	(0.17)	(0.18)	(0.28)	(0.09)	(0.13)	(0.09)	(0.13)	(0.27)	(0.32)	(0.88)
EGFR_CKD-EPI_ (ml/min/1.73 m^2^)	133	112	124	109	87	96	79	59	63	50	29	22
	(0.36)	(0.51)	(0.14)	(0.12)	(0.27)	(0.18)	(0.20)	(0.13)	(0.20)	(0.30)	(0.24)	(0.92)
Age (years)	26 (0.2)	53 (0.8)	28 (0.3)	39 (0.2)	79 (0.5)	40 (0.5)	59 (0.6)	83 (1.0)	56 (1.11)	72 (0.74)	81 (2.12)	72 (1.75)
Age < 65 years, N (%) ^a b^	2,415	247	1,473	4,614	0	1,754	1,717	0	100	115	0	12
	(99.9)	(85.8)	(100)	(96.4)		(95.8)	(61.3)		(83.8)	(18.0)		(16.9)
Male (%)	40	55	44	53	56	46	49	60	24	40	72	35
Race/Ethnicity (%)												
Non-Hispanic White	52	67	65	74	85	86	86	86	90	86	90	73
Non-Hispanic Black	25	9	19	11	7	4	7	13	1	8	9	18
Mexican American	13	10	8	6	2	4	2	1	2	1	1	2
Other	11	13	9	10	7	6	6	1	6	5	0	8
Ever or Current Smoker (%)	45	68	46	56	47	51	54	43	57	55	84	56
Diabetes (%)	3	13	<1	3	11	3	8	6	14	15	27	26
Prior CVD (%)	<1	<1	<1	3	16	3	10	22	13	26	73	35
Body Mass Index (kg/m^2^)	25	27	25	26	25	26	27	25	29	28	25	26
	(0.23)	(0.75)	(0.20)	(0.14)	(0.26)	(0.20)	(0.15)	(0.45)	(0.68)	(0.24)	(1.02)	(1.18)
Systolic Blood Pressure, mm Hg	112	130	112	119	145	118	131	143	130	143	150	145
	(0.46)	(1.90)	(0.34)	(0.31)	(1.86)	(0.45)	(0.59)	(1.99)	(2.22)	(1.03)	(6.01)	(3.66)
Diastolic Blood Pressure, mm Hg	68	78	69	74	73	75	76	72	76	75	71	73
	(0.41)	(0.95)	(0.35)	(0.28)	(1.05)	(0.32)	(0.25)	(1.14)	(0.85)	(0.49)	(2.11)	(2.98)
Antihypertensive medication use (%)	4	26	2	10	32	10	27	44	36	60	75	74
Total Cholesterol (mg/dl)	183	217	181	198	208	204	217	213	230	232	209	230
	(1.69)	(4.92)	(1.68)	(1.14)	(3.82)	(1.57)	(1.21)	(5.49)	(4.65)	(2.81)	(10.31)	(14.24)
CRP (mg/dl; %)												
<0.22	73	58	80	75	71	76	67	62	64	52	29	42
0.22-0.99	19	29	14	19	22	19	25	28	23	32	12	40
≥1.00	9	13	6	6	7	5	8	10	13	16	59	18
ACR (mg/g; %)												
<30	93	84	93	94	74	95	89	78	83	70	9	34
30-300	6	11	7	5	25	5	10	18	15	23	68	20
≥300	<1	5	<1	<1	17	1	1	4	3	7	23	46

Abbreviations: *CVD*, Cardiovascular Disease; *eGFR*, estimated glomerular filtration rate; *MDRD*, Modification of Diet in Renal Disease Study; *CKD-EPI*, Chronic Kidney Disease Epidemiology Collaboration; *CRP*, C-reactive protein; *ACR*, albumin-to-creatinine ratio.

Note: Conversion factors for units: low- and high-density lipoprotein cholesterol in mg/dL to mmol/L, ×0.02586; triglycerides in mg/dL to mmol/L, ×0.01129; creatinine in mg/dL to μmol/L, ×88.4; eGFR in mL/min/1.73 m2 to mL/s/1.73 m2, ×0.01667; albumin-creatinine ratio in mg/g to mg/mmol, divide by 8.84.

^a^ Participant numbers are unweighted N.

^b^ Percent represents the population percent representative of the non-institutionalized US population; total may not equal 100% due to rounding.

^c^ Values presented are population % for categorical variables and mean (linearized standard error) for continuous variables.

^d^ One 86 year old Mexican-American female with serum creatinine = 0.488 mg/dL had eGFR_MDRD_ = 120 ml/min/1.73 m^2^ and eGFR_CKD-EPI_ 89 ml/min/1.73 m^2^ (data not shown).

^e^ One 64 year old Mexican-American female with serum creatinine = 1.736 had eGFR_MDRD_ = 29.5 ml/min/1.73 m^2^ and eGFR_CKD-EPI_ = 30.6 ml/min/1.73 m^2^ (data not shown).

### Estimated GFR and the risk of death

**Figure 1:**
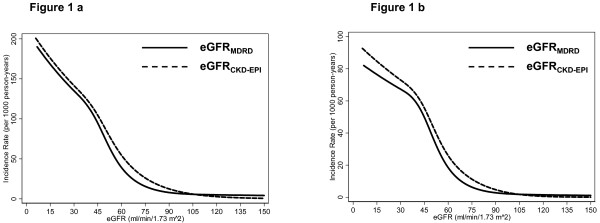
**Unadjusted Incidence Rates of Mortality with eGFR**_**MDRD**_**and eGFR**_**CKD-EPI**_**in the US Population: NHANES III (1988–1994).a**: All-Cause Mortality. **b**: Cardiovascular Disease Mortality.

There were 3,620 deaths over 215,082 person-years of follow-up (median, 14.3 years). The weighted unadjusted incidence rate for all-cause mortality was higher for eGFR_CKD-EPI_ compared with eGFR_MDRD_ below 90 ml/min/1.73 m^2^ (Figure1a). Within categories of eGFR, the hazards for all-cause mortality adjusted for demographic characteristics, comorbidities, CRP and ACR were higher for eGFR_CKD-EPI_ categories compared with eGFR_MDRD_ categories (Table2). With both eGFR equations, there was U-shaped association with mortality with higher risk of death for eGFR above 120 ml/min/1.73 m^2^ and below 60 ml/min/1.73 m^2^ compared with eGFR 90–119 ml/min/1.73 m^2^. There were few individuals with eGFR <30 ml/min/1.73 m^2^ (eGFR_MDRD_, n = 56; eGFR_CKD-EPI_, n = 67) and few deaths in individuals with eGFR ≥120 ml/min/1.73 m^2^ (eGFR_MDRD_, n = 228; eGFR_CKD-EPI_, n = 179). Overall, the trends for association between eGFR categories and CVD mortality (n = 1,540) were very similar to all-cause mortality (Figure1b and Table2).

**Table 2:** **Adjusted**^**a**^**hazard ratio (95% Confidence interval) of all-Cause and cardiovascular disease mortality, by eGFR categories among 16,010 participants of NHANES III (1988–1994) with follow-up till December 31, 2006**

		**Categories of eGFR (ml/min/1.73 m**^**2**^**)**				
		**≥ 120**	**90-119**	**60-89**	**30-59**	**< 30**
**All-Cause Mortality**						
**EGFR**_**CKD-EPI**_	Participants, N	3,889	7,151	3,947	956	67
	Deaths, N	179	921	1,686	775	59
	HR	2.05	Reference	0.97	1.39	1.38
	(95% CI)	(1.55-2.71)		(0.86-1.10)	(1.17-1.65)	(0.88-2.16)
**EGFR**_**MDRD**_	Participants, N	2,729	6,604	5,606	1,015	56
	Deaths, N	228	805	1,788	750	49
	HR	1.70	Reference	0.94	1.31	1.96
	(95% CI)	(1.36-2.14)		(0.84-1.05)	(1.11-1.56)	(1.11-3.44)
**CVD Mortality**						
**EGFR**_**CKD-EPI**_	Participants, N	3,889	7,151	3,947	956	67
	Deaths, N	41	303	755	412	29
	HR	2.70	Reference	1.05	1.49	1.64
	(95% CI)	(1.54-4.73)		(0.87-1.26)	(1.16-1.92)	(1.02-2.65)
**EGFR**_**MDRD**_	Participants, N	2,729	6,604	5,606	1,015	56
	Deaths, N	64	278	774	401	23
	HR	1.53	Reference	0.95	1.32	2.17
	(95% CI)	(0.96-2.45)		(0.79-1.13)	(0.99-1.78)	(1.18-3.98)

Abbreviations: *eGFR*, estimated glomerular filtration rate; *MDRD*, Modification of Diet in Renal Disease Study; *CKD-EPI*, Chronic Kidney Disease Epidemiology Collaboration; *CVD*, Cardiovascular Disease.

^a^ Adjusted for age, sex, race/ethnicity, prior CVD, diabetes, smoking, systolic and diastolic blood pressure, use of antihypertensive medications, body mass index, cholesterol, C-reactive protein category and albumin-to-creatinine ratio category.

### Reclassification by eGFR_CKD-EPI_ and the risk of death

**Figure 2:**
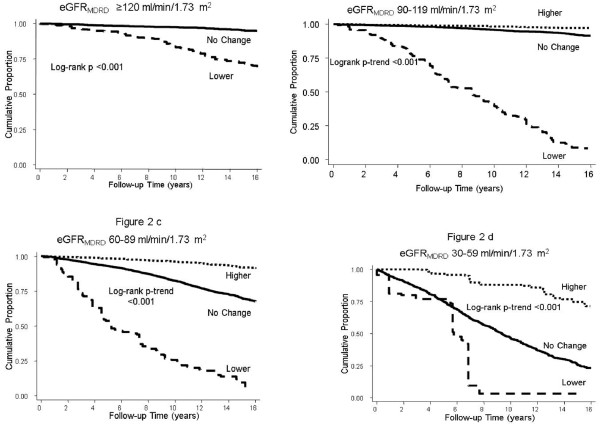
**Cumulative Incidence of All-Cause Mortality with Reclassification by eGFR**_**CKD-EPI**_**within eGFR**_**MDRD**_**Categories in the US Population: NHANES III (1988–1994).a**: Reclassification within eGFR_MDRD_ Category ≥120 ml/min/1.73 m^2^. **b**: Reclassification within eGFR_MDRD_ Category 90–119 ml/min/1.73 m^2^. **c**: Reclassification within eGFR_MDRD_ Category 60–89 ml/min/1.73 m^2^. **d**: Reclassification within eGFR_MDRD_ Category 30–59 ml/min/1.73 m^2^.

Figure2 displays the unadjusted cumulative incidence of death with reclassification by eGFR_CKD-EPI_. Those classified upwards to a higher eGFR_CKD-EPI_ category had lower cumulative incidence of mortality while those reclassified downward to lower eGFR_CKD-EPI_ category had higher cumulative incidence of death compared with those not reclassified. Overall, compared with no reclassification, the hazard ratio (HR) of all-cause mortality with reclassification to a higher eGFR_CKD-EPI_ category was 0.34 (95% CI, 0.28-0.41) and with reclassification to a lower eGFR_CKD-EPI_ category was 3.56 (95% CI, 3.04-4.16). After adjustment for age, sex and race/ethnicity, the HR was 0.91 (95% CI, 0.75-1.10) and 1.28 (95% CI, 1.11-1.48) for reclassification to a higher and lower eGFR_CKD-EPI_ category, respectively.

Table3 displays the adjusted HR for all-cause and CVD mortality with reclassification by eGFR_CKD-EPI_ within categories of eGFR_MDRD_. Among those classified as eGFR_MDRD_ 30–59 ml/min/1.73 m^2^, reclassification to a higher eGFR_CKD-EPI_ category was associated with a 47% lower hazard of death compared with those not reclassified (HR adjusted for demographic characteristics, comorbidities, CRP and albuminuria, 0.53; 95% CI, 0.34-0.84). There were very few individuals (n = 12) with eGFR_MDRD_ 30–59 ml/min/1.73 m^2^ who were reclassified to a lower eGFR_CKD-EPI_ category.

**Table 3:** **Reclassification and adjusted hazard ratios**^**a**^**of all-cause and cardiovascular disease mortality by eGFR categories determined using the MDRD and the CKD-EPI study equation: NHANES III (1988–1994) - follow-up till December 31, 2006**

	**EGFR**_**CKD-EPI**_**Categories**				
**EGFR**_**MDRD**_**Categories**	>120	90-119	60-89	30-59	<30
**>120**					
Reclassified, N ^b^ (%)^c^	2,416 (88.3)	312 (11.7)			
**All-Cause Mortality**					
Deaths, N ^b^	126	101			
HR (95% CI)	REFERENCE	0.57 (0.26-1.26)			
**CVD Mortality**					
Deaths ^b^	28	36			
HR (95% CI)	REFERENCE	0.37 (0.15-0.94)			
**90-119**					
Reclassified, N ^b^ (%)^c^	1,473 (20.2)	4,969 (78.4)	162 (1.4)		
**All-Cause Mortality**					
Deaths, N ^b^	53	604	148		
HR (95% CI)	1.42 (0.85-2.37)	REFERENCE	1.39 (0.94-2.07)		
**CVD Mortality**					
Deaths ^b^	13	193	72		
HR (95% CI)	2.95 (1.03-8.42)	REFERENCE	1.79 (0.94-3.39)		
**60-89**					
Reclassified, N ^b^ (%)^c^		1,870 (42.9)	3,664 (56.6)	72 (0.5)	
**All-Cause Mortality**					
Deaths, N ^b^		216	1,506	66	
HR (95% CI)		1.13 (0.91-1.42)	REFERENCE	1.31 (1.01-1.69)	
**CVD Mortality**					
Deaths ^b^		74	669	31	
HR (95% CI)		1.02 (0.69-1.50)	REFERENCE	1.42 (1.01-1.99)	
**30-59**					
Reclassified, N ^b^ (%)^c^			120 (19.4)	883 (79.5)	12 (1.1) ^d^
**All-Cause Mortality**					
Deaths, N ^b^			31	708	11
HR (95% CI)			0.53 (0.34-0.84)	REFERENCE	N/A
**CVD Mortality**					
Deaths ^b^			14	381	6
HR (95% CI)			0.51(0.27-0.96)	REFERENCE	N/A

Abbreviations: *eGFR*, estimated glomerular filtration rate; *MDRD*, Modification of Diet in Renal Disease Study; *CKD-EPI*, Chronic Kidney Disease Epidemiology Collaboration; *HR*, Hazard Rate; *CVD*, cardiovascular disease; *N/A*, not applicable.

^a^ Adjusted for age, sex, race/ethnicity, prior CVD, systolic or diastolic blood pressure, use of antihypertensive medications, diabetes status, smoking status, body mass index, cholesterol, CRP category and albumin-to-creatinine ratio category.

^b^ Numbers represent crude (un-weighted) participant number.

^c^ Percent represents the population percent representative of the non-institutionalized US population; total may not equal 100% due to rounding.

^d^ HR not presented as there are less than 30 people in the cell.

Those who were reclassified upwards to eGFR_CKD-EPI_ ≥60 ml/min/1.73 m^2^ from eGFR_MDRD_ 30–59 ml/min/1.73 m^2^ had similar risk of all-cause mortality as those with eGFR ≥60 ml/min/1.73 m^2^ by both equations (adjusted HR, 1.05; 95% CI, 0.62-1.77). Reclassification from eGFR_MDRD_ category 60–89 ml/min/1.73 m^2^ to a lower eGFR_CKD-EPI_ category (30–59 ml/min/1.73 m^2^) was associated with a higher risk of death (adjusted HR, 1.31; 95% CI, 1.01-1.69) compared with those not reclassified. There were no significant differences noted in all-cause mortality among individuals reclassified in eGFR_CKD-EPI_ categories 90–119 and ≥120 ml/min/1.73 m^2^. Very similar trends were seen with CVD mortality.

### Net reclassification improvement by eGFR_CKD-EPI_

To evaluate the effect of reclassification on mortality, we restricted our analysis to individuals with eGFR_MDRD_ and eGFR_CKD-EPI_ <120 ml/min/1.73 m^2^. Table4 presents the NRI for all-cause and CVD mortality. The overall NRI for eGFR_CKD-EPI_ for all-cause mortality was 0.21 (p < 0.001) and for CVD mortality was 0.22 (p < 0.001). In age stratified analyses, the NRIs was lower as expected but remained substantial for participant age >65 years (0.14 for all-cause and 0.09 for CVD, p < 0.001). The NRI was also significant stratified by sex and most ethnicity groups as well as stratified by sex and limited to older participants (Additional file 1 Table S1**,** NRI 0.09 for men and 0.15 for women age >65 years).

**Table 4:** **Net reclassification improvement by the CKD-EPI equation among participants with eGFR <120 ml/min/1.73 m**^**2**^**by both equations stratified by age, sex and race**

	**Reclassification, number (Population %)**^**a**^	**Deaths,**	**NRI**	
		**All-cause (CVD)**	**All-cause**	**CVD**
**Overall**	11,808 (24.1%)	3,339 (1,463)	0.2073***	0.2216***
**By Age Categories**				
17-44	4,822 (30.3%)	208 (58)	0.0216	−0.0883
45-64	3,491 (21.8%)	715 (258)	−0.0146	−0.0006
≥65	3,495 (11.4%)	2,416 (1,147)	0.1362***	0.0943***
**By Sex**				
Male	5,839 (22.1%)	1,844 (796)	0.2077***	0.2277***
Females	5,969 (26.0%)	1,495 (667)	0.2063***	0.2157***
**By Race/Ethnicity**				
NH Whites	5,736 (25.7%)	1,957 (903)	0.2258***	0.2368***
NH Blacks	2,701 (12.8%)	710 (290)	0.1245***	0.1229***
Mex-Am	2,868 (20.2%)	594 (239)	0.0648**	0.0682*
Others	503 (20.2%)	78 (31)	0.1427*	0.2559*** ^b^

Abbreviations: *eGFR*, estimated glomerular filtration rate; *MDRD*, Modification of Diet in Renal Disease Study; *CKD-EPI*, Chronic Kidney Disease Epidemiology Collaboration; *NRI*, Net Reclassification Improvement; *NH*, Non-Hispanic; *Mex-Am*, Mexican-American.

^a^ Reclassification to a different eGFR category by CKD-EPI equation. Number represents the number of participants; Population % is representative of the non-institutionalized US population.

^b^ NRI estimates less precise as there are <50 events in this cell.

* p < 0.05; ** p < 0.01; *** p < 0.001.

## Discussion

In this study of a representative sample of US adults during 18 years of follow-up, eGFR_CKD-EPI_ improved risk stratification. Among those classified as eGFR_MDRD_ 30–59 ml/min/1.73 m^2^, 19.4% were reclassified to eGFR >60 ml/min/1.73 m^2^ by the CKD-EPI equation and this upward reclassification was associated with 47% lower risk of all-cause mortality and 49% lower risk of CVD mortality compared with individuals with eGFR 30–59 ml/min/1.73 m^2^ by both the MDRD Study and CKD-EPI equations. Among those with eGFR_MDRD_ 60–89 ml/min/1.73 m^2^, 0.5% were reclassified downwards to eGFR 30–59 ml/min/1.73 m^2^ by the CKD-EPI equation and this downward classification was associated with 31% higher risk of all-cause mortality and 42% higher risk of CVD mortality. Overall, CKD-EPI equation significantly improved risk prediction for both all-cause and CVD mortality. The better risk categorization by the CKD-EPI equation was observed particularly in those older than 65 years at baseline.

CKD-EPI equation improves GFR estimation compared with the MDRD Study equation and eGFR_CKD-EPI_ is significantly more accurate than eGFR_MDRD_ above and below 60 ml/min/1.73 m^2^ as well as across racial and ethnic subgroups [2,13]. The properties of the CKD-EPI equation result in higher eGFR in younger individuals, whites and females. In NHANES, we noted that reclassification moved individuals with higher comorbidities to a lower eGFR_CKD-EPI_ category and individuals with lower comorbidities to higher eGFR_CKD-EPI_ categories. In the clinically important CKD stage 3 (eGFR_MDRD_ 30–59 ml/min/1.73 m^2^), almost 20% of the population was reclassified to a higher eGFR_CKD-EPI_ category. In unadjusted and adjusted analyses, this reclassification was associated with a lower risk of death, suggesting that eGFR_CKD-EPI_ in this range may have clinical significance even without information about other comorbidities. These findings have important implications for both individual clinical risk stratification and screening.

A number of recent studies have demonstrated that the use of eGFR_CKD-EPI_ results in improvement in risk classification of individuals. In the Australian Diabetes, Obesity and Lifestyle (AusDiab) Study, 25% of the participants with eGFR_MDRD_ <60 ml/min/1.73 m^2^ were reclassified to higher eGFR_CKD-EPI_ category [4]. The risk of death in individuals with eGFR_MDRD_ <60 ml/min/1.73 m^2^ reclassified to eGFR >60 ml/min/1.73 m^2^ by the CKD-EPI equation was similar to those with eGFR_MDRD_ ≥60 ml/min/1.73 m^2^ (HR, 1.01; 95% CI, 0.62-1.97). In the Atherosclerosis Risk in Communities (ARIC) Study, a prospective cohort of 45–64 year old whites and African-Americans from 4 US communities (N = 13,905), reclassification from eGFR_MDRD_ 30–59 ml/min/1.73 m^2^ to eGFR_CKD-EPI_ 60–89 ml/min/1.73 m^2^ was associated with a lower risk of all-cause mortality in the unadjusted models but was no longer significant after adjustment for age, sex and race. NRI for all-cause mortality was 0.095 (p < 0.001) [3]. Among participants of Kidney Early Evaluation Program (KEEP), a US screening program for individuals at high risk of chronic kidney disease, 17.5% were reclassified to higher eGFR categories and 2.7% to lower categories. Upward reclassification was associated with lower mortality, downward reclassification was associated with higher mortality and NRI was 0.159 [6]. In a post hoc analysis of the VALIANT trial, eGFR_CKD-EPI_ categories improved risk stratification and the NRI for the composite end point of CVD death, recurrent myocardial infarction, heart failure, or stroke was 0.087 [5]. Our study extends these findings in a study with results generalizable to the US population and quantifies the improvement in risk stratification in African-Americans and Mexican-Americans.

We used NRI to assess the improvement in risk prediction with CKD-EPI equation compared with the MDRD Study equation. The improvement in risk prediction is expected to be relatively small as both equations use the same variables and have the inherent limitation of serum creatinine as a marker of GFR. Traditional methods for risk prediction, such as area under the receiver-operating-characteristic curve (AUC) require large independent associations, often over 2–3 fold, to result in meaningful improvement in AUC [14-16]. The NRI is particularly well suited when categories are associated with clinical action as is the case for estimated GFR. The NRI was lower in younger age groups despite high reclassification rates, possibly since death rates are low at younger age. Among older age groups (>65 years), NRIs were quite high (0.14 for all-cause and 0.09 for CVD mortality) despite reclassification being lower (11% compared to >30% in younger ages). Notably, the NRIs in the older age group remain significant even after stratification with sex or race/ethnicity. In contrast to ARIC and KEEP, NRIs in NHANES were not positive or statistically significant in those 45–64 years of age. These discrepant findings require further study across a range of populations.

We noted increased risk of all-cause and CVD death among individuals with eGFR ≥120 ml/min/1.73 m^2^ by either equation compared with eGFR 90–119 ml/min/1.73 m^2^ after the adjustment for potential confounders (Table2). The HR was higher for eGFR_CKD-EPI_ than for eGFR_MDRD_ in this category. This association has been noticed in previous studies and replicates the well-known U- shaped relationship between serum creatinine and mortality. This U-shape likely includes components of low muscle mass and hyperfiltration which cannot be separated using only serum creatinine as a marker of GFR. They persist despite the inclusion of a spline term for serum creatinine in the CKD-EPI equation which reduces the very high eGFRs calculated using the MDRD Study equation.

The strengths of our study includes its large sample size, prospective design, large sample of racial/ethnic minorities, broad age range of the population, rigorous data collection and extensive information on covariates, prior work to standardize serum creatinine, measurement of ACR and CRP, near-complete mortality follow-up using the NDI and large number of events during the follow-up period. The results of our study are generalizable to the non-institutionalized population of the U.S. Some limitations of our study also deserve mention. GFR was not measured but was estimated using serum creatinine and not all participants were fasting prior to serum creatinine measurement. Nonetheless, serum creatinine and estimated GFR are routinely used measures of kidney function in clinical practice and our data reflect common clinical information. We had relatively few individuals with eGFR_MDRD_ <30 ml/min/1.73 m^2^. Cause of death was ascertained *via* NDI linkage of NHANES III data and not independently adjudicated and there is potential of misclassification of CVD mortality. Importantly, we did not have information about kidney failure requiring renal replacement therapy, an important outcome that deserves examination in future studies.

## Conclusion

The CKD-EPI equation for estimating GFR predicts risk at least as well as the MDRD Study equation in the general US population and improves risk classification of individuals, particularly among those older than 65 years. Our data, in conjunction with previously reported studies, suggest that adoption of CKD-EPI equation for eGFR reporting may improve clinical risk stratification.

## Competing interests

The authors declare that they have no competing interests.

## Authors’ contributions

TS, KM, LA and JC developed the study design. TS performed the statistical analyses and KM, BA, ES and YS assisted with statistical analyses and interpretation of data. TS drafted the manuscript and all authors read and approved the manuscript.

### Disclosure

Dr. Shafi was supported by K23-DK-083514. Dr. Selvin was supported by K01-DK-076595. Drs. Astor and Coresh are supported by R01-DK-076770. Dr. Stevens is supported by K23-DK-081017.

## Pre-publication history

The pre-publication history for this paper can be accessed here:

http://www.biomedcentral.com/1471-2369/13/42/prepub

## Supplementary Material

Additional file 1 **Table S1.** Net Reclassification Improvement by the CKD-EPI Equation among Participants with eGFR <120 ml/min/1.73 m^2^ by both equations stratified by Sex, Age and Race. Click here for file

## References

[B1] Levey AS Stevens LA Estimating GFR using the CKD Epidemiology Collaboration (CKD-EPI) creatinine equation: more accurate GFR estimates, lower CKD prevalence estimates, and better risk predictions Am J Kidney Dis 2010 55 4 622 627 10.1053/j.ajkd.2010.02.337 20338463 PMC2846308

[B2] Levey AS Stevens LA Schmid CH Zhang YL Castro AF Feldman HI Kusek JW Eggers P Van Lente F Greene T A new equation to estimate glomerular filtration rate Ann Intern Med 2009 150 9 604 612 19414839 10.7326/0003-4819-150-9-200905050-00006 PMC2763564

[B3] Matsushita K Selvin E Bash LD Astor BC Coresh J Risk implications of the new CKD Epidemiology Collaboration (CKD-EPI) equation compared with the MDRD Study equation for estimated GFR: the Atherosclerosis Risk in Communities (ARIC) Study Am J Kidney Dis 2010 55 4 648 659 10.1053/j.ajkd.2009.12.016 20189275 PMC2858455

[B4] White SL Polkinghorne KR Atkins RC Chadban SJ Comparison of the prevalence and mortality risk of CKD in Australia using the CKD Epidemiology Collaboration (CKD-EPI) and Modification of Diet in Renal Disease (MDRD) Study GFR estimating equations: the AusDiab (Australian Diabetes, Obesity and Lifestyle) Study Am J Kidney Dis 2010 55 4 660 670 10.1053/j.ajkd.2009.12.011 20138414

[B5] Skali H Uno H Levey AS Inker LA Pfeffer MA Solomon SD Prognostic assessment of estimated glomerular filtration rate by the new Chronic Kidney Disease Epidemiology Collaboration equation in comparison with the Modification of Diet in Renal Disease Study equation Am Heart J 2011 162 3 548 554 10.1016/j.ahj.2011.06.006 21884875

[B6] Stevens LA Li S Kurella Tamura M Chen SC Vassalotti JA Norris KC Whaley-Connell AT Bakris GL McCullough PA Comparison of the CKD Epidemiology Collaboration (CKD-EPI) and Modification of Diet in Renal Disease (MDRD) study equations: risk factors for and complications of CKD and mortality in the Kidney Early Evaluation Program (KEEP) Am J Kidney Dis 2011 57 3 Suppl 2 S9 S16 21338849 10.1053/j.ajkd.2010.11.007 PMC3298760

[B7] Centers for Disease Control and Prevention (CDC). National Center for Health Statistics (NCHS) National Health and Nutrition Examination Survey Data Department of Health and Human Services, Centers for Disease Control and Prevention, Hyattsville [http://www.cdc.gov/nchs/nhanes/nh3data.htm]. Last accessed May 16, 2012

[B8] Selvin E Manzi J Stevens LA Van Lente F Lacher DA Levey AS Coresh J Calibration of serum creatinine in the National Health and Nutrition Examination Surveys (NHANES) 1988–1994, 1999–2004 Am J Kidney Dis 2007 50 6 918 926 10.1053/j.ajkd.2007.08.020 18037092

[B9] Levey AS Bosch JP Lewis JB Greene T Rogers N Roth D A more accurate method to estimate glomerular filtration rate from serum creatinine: a new prediction equation Modification of Diet in Renal Disease Study Group. Ann Intern Med 1999 130 6 461 470 10.7326/0003-4819-130-6-199903160-00002 10075613

[B10] National Center for Health Statistics Office of Analysis and Epidemiology, Public-use Third National Health and Nutrition Examination Survey Linked Mortality File 2010, Hyattsville [Available at the following address: http://www.cdc.gov/nchs/data_access/data_linkage/mortality/nhanes3_linkage_public_use.htm]

[B11] National Center for Health Statistics Office of Analysis and Epidemiology, The Third National Health and Nutrition Examination Survey (NHANES III) Linked Mortality File, Mortality follow-up through 2006 2006 Matching Methodology May 2009, Hyattsville Available at the following address: http://www.cdc.gov/nchs/data/datalinkage/matching_methodology_nhanes3_final.pdf. Last accessed May 16, 2012

[B12] Pencina MJ D'Agostino RB D'Agostino RB Vasan RS Evaluating the added predictive ability of a new marker: from area under the ROC curve to reclassification and beyond Stat Med 2008 27 2 157 172 discussion 207–112 10.1002/sim.2929 17569110

[B13] Stevens LA Schmid CH Greene T Zhang YL Beck GJ Froissart M Hamm LL Lewis JB Mauer M Navis GJ Comparative performance of the CKD Epidemiology Collaboration (CKD-EPI) and the Modification of Diet in Renal Disease (MDRD) Study equations for estimating GFR levels above 60 mL/min/1.73 m2 Am J Kidney Dis 2010 56 3 486 495 10.1053/j.ajkd.2010.03.026 20557989 PMC2926290

[B14] Greenland P O'Malley PG When is a new prediction marker useful? A consideration of lipoprotein-associated phospholipase A2 and C-reactive protein for stroke risk Arch Intern Med 2005 165 21 2454 2456 10.1001/archinte.165.21.2454 16314539

[B15] Pepe MS Janes H Longton G Leisenring W Newcomb P Limitations of the odds ratio in gauging the performance of a diagnostic, prognostic, or screening marker Am J Epidemiol 2004 159 9 882 890 10.1093/aje/kwh101 15105181

[B16] Ware JH The limitations of risk factors as prognostic tools N Engl J Med 2006 355 25 2615 2617 10.1056/NEJMp068249 17182986

