# Supplementary material for: Comparing the association of GFR estimated by the CKD-EPI and MDRD study equations and mortality: the third national health and nutrition examination survey (NHANES III)
Source: BMC Nephrol. 2012 Jun 15;13:42. doi: 10.1186/1471-2369-13-42 (PMC3447668; doi:10.1186/1471-2369-13-42)
Supplement: Additional file 1 — Table S1. Net Reclassification Improvement by the CKD-EPI Equation among Participants with eGFR <120 ml/min/1.73 m2 by both equations stratified by Sex, Age and Race. [file 1471-2369-13-42-S1.doc]

**Supplemental Table A. Net Reclassification Improvement by the CKD-EPI Equation among Participants with eGFR <120 ml/min/1.73 m^2^ by both equations stratified by Sex, Age and Race**

|  | **Men** | | | | | **Women** | | | | |
| --- | --- | --- | --- | --- | --- | --- | --- | --- | --- | --- |
|  |  |  | Deaths,  All-Cause (CVD) | NRI | |  |  | Deaths,  All-Cause (CVD) | NRI | |
|  | Reclassification,  Number (Population %) ^a^ | |  | All-Cause | CVD | Reclassification, Number (Population %) ^a^ | |  | All-Cause | CVD |
| **Overall** | 5,839 (22.1%) | | 1,844 (796) | 0.2077*** | 0.2277*** | 5,969 (26.0%) | | 1,495 (667) | 0.2063*** | 0.2157*** |
| **By Age Categories** | | | | | | | | | | |
| 17-44 | 2,413 (27.0%) | | 130 (43) | 0.0960 | 0.0540  ^b^ | 2,409 (33.9%) | | 78 (15) | -0.0960 | -0.3433*  ^b^ |
| 45-64 | 1,691 (20.5%) | | 403 (159) | -0.0107 | 0.0219 | 1,800 (23.0%) | | 312 (99) | -0.0218 | -0.0365 |
| >65 | 1,735 (9.9%) | | 1,311 (594) | 0.0922*** | 0.0776** | 1,760 (12.6%) | | 1,105 (553) | 0.1534*** | 0.1026*** |
| **By Race/Ethnicity** | | | | | | | | | | |
| NH Whites | 2,732 (22.9%) | | 1,045 (478) | 0.2112*** | 0.2324*** | 3,004 (28.5%) | | 912 (425) | 0.2398*** | 0.2414*** |
| NH Blacks | 1,330 (12.0%) | | 403 (161) | 0.1354*** | 0.1273*** | 1,371 (13.5%) | | 307 (129) | 0.1110*** | 0.1164*** |
| Mex-Am | 1,554 (20.1%) | | 358 (142) | 0.0683* | 0.0169 | 1,314 (20.3%) | | 136 (97) | 0.0597 | 0.1423** |
| Others | 223 (25.7%) | | 38 (15) | 0.3168***  ^b^ | 0.4497***  ^b^ | 280 (14.5%) | | 40 (16) | -0.0480  ^b^ | 0.0428  ^b^ |

Abbreviations: eGFR, estimated glomerular filtration rate; MDRD, Modification of Diet in Renal Disease Study; CKD-EPI, Chronic Kidney Disease Epidemiology Collaboration; NRI, Net Reclassification Improvement; NH, Non-Hispanic; Mex-Am, Mexican-American

^a^ Reclassification to a different eGFR category by CKD-EPI equation. Number represents the number of participants; Population % is representative of the non-institutionalized US population.

^b^ NRI estimates less precise as there are <50 events in this cell.

* p<0.05; ** p<0.01; *** p<0.001
